# Three-dimensional assessment of two different canine retraction techniques: a randomized split-mouth clinical trial

**DOI:** 10.1186/s40510-021-00374-4

**Published:** 2021-08-09

**Authors:** Şuayip Akın, Hasan Camcı

**Affiliations:** Department of Orthodontics, Afyonkarahisar Health Science University, Afyonkarahisar, Turkey

**Keywords:** Canine retraction, Bodily movement, Sliding mechanics

## Abstract

**Introduction:**

The aim of this split-mouth trial was to compare power-arm sliding (PAS) and direct sliding (DS) canine retraction mechanics in terms of speed, rotation, angulation, and anchorage loss.

**Methods:**

Thirty-six class II division 1 patients (20 females, 16 males; mean age, 16.94 ± 3.23) requiring upper first premolar extraction were included in the study. Miniscrews were used as anchorage units, and a retraction force of 150 gr was applied from the power arm on one side and from the bracket on the opposite side by using elastomeric chains. Randomization was achieved by block randomization with a 1:1 allocation ratio either to the right or the left with allocations concealed in opaque, sealed envelopes. Digital models were acquired using an intraoral scanner at the beginning of the retraction (T0), the first month (T1), the second month (T2), and the third month (T3). Before the scans, the archwire was removed, and custom metal jigs were inserted into the vertical slot of the canine brackets to evaluate the canine angulation. The digital models of each patient were separately superimposed with the local best-fit algorithm, and the retraction rate, angulation, rotation, and anchorage loss were measured. The digital measurements were performed using the Geomagic Control X software.

**Results:**

The DS technique’s total retraction rate was higher than that of the PAS technique (2.09 and 1.57, respectively, *p* = .002). There was, however, no significant difference between the two techniques in terms of angulation, rotation, and anchorage loss. A negative correlation was observed between the retraction rate and age, but it was not statistically significant. No significant difference was observed between the retraction rates of female and male participants in either retraction technique.

**Conclusions:**

For both orthodontists and patients, the DS technique is simpler and more convenient; thus, it is the preferred method for canine retraction.

**Trial registration:**

The trial was not registered.

**Protocol:**

The protocol was not published before the trial commencement.

## Introduction and literature review

The extraction decision is included in the treatment plan for approximately 50% of orthodontic cases, and the upper first premolar is one of the most frequently extracted teeth [1]. Canines are moved to the extraction space using a wide variety of mechanics after the extraction of premolars [2]. Canine retraction takes an average of 6–9 months [3], and that constitutes an important part of treatment time. Orthodontists desire a rapid, accurate (bodily movement and without rotation) retraction of the canines. Dozens of frictional or non-frictional mechanics have been developed to meet the demand for them in contemporary orthodontics [4]. In frictional mechanics, the retraction force can be directly applied from the canine bracket (direct sliding (DS)) or the power arm (power arm sliding (PAS)) [5].

Depending on the size and location of the force applied during retraction, canine teeth might exhibit different types and rates of movement. The retraction force should pass through the center of resistance for bodily movement; otherwise, a tipping movement could occur [6].

Post-treatment tooth movement evaluation is generally performed using cephalometric x-rays [3], panoramic radiographs [7], or plaster models [8]. In recent years, the use of three-dimensional digital models as an alternative to traditional methods has become widespread in the assessment of tooth movements [9, 10]. A number of studies have reported that digital models show high accuracy and repeatability in orthodontic model analysis [11, 12].

Stainless steel or titanium miniscrews of various sizes and diameters are widely used in modern orthodontics [13, 14]. They can be used as indirect anchoring units to prevent the loss of anchorage or as direct anchorage units to allow for tooth movement. Miniscrews have become popular due to the simplification of orthodontic biomechanics and easy insertion and removal. In canine retraction cases, miniscrews (i.e., temporary anchorage devices) are often used for these mechanical advantages.

### Specific objectives and hypothesis

The purpose of this study was to compare the effects (rate, rotation, angulation, and anchorage loss) of different force application methods (below or close to the center of resistance) in canine retraction by using three-dimensional digital model measurements. It was also to determine whether there was a correlation between age, gender, and other variables. The null hypothesis of this study was that there is no difference in DS and PAS frictional mechanics in terms of rate, rotation, angulation, and anchorage loss.

## Materials and methods

### Trial design and any changes after trial

The current study was a split-mouth randomized clinical trial with a 1:1 allocation. After the trial began, the methods remained unchanged.

### Participants, eligibility criteria, and settings

The experimental protocol of this prospective study was approved by the Clinical Research Ethics Committee of Afyonkarahisar Health Science University (ID:103/06.03.2020). Informed consent forms were obtained from all participants or their legal guardians. Inclusion criteria were no previous orthodontic or periodontal treatment history, no bone loss, no systemic diseases, no routinely used drugs, no smoking, good oral hygiene, and a C5 or C6 cervical vertebra maturation phase. Exclusion criteria were class I and class III malocclusions, severe skeletal class II (overjet > 10 mm), and the long-term use of drugs such as anti-inflammatories, systemic corticosteroids, and antibiotics. The average amount of crowding for the patients was 3.37±3.21.

### Sample size calculation

The sample size calculation using the GPower software revealed that at least 28 patients were required (effect size = 0.8, *α* = 0.05, and 1-*β* = 0.90) [15]. The study was conducted on 37 patients who required upper first premolar extraction (20 females, 16 males; mean age, 16.94 ± 3.23 years).

### Randomization

The power arm application was randomly selected either to the right or left side with a 1:1 allocation ratio using sealed, opaque envelopes. To avoid selection bias, each patient was requested to select previously shuffled envelopes, which also protected the assignment sequence during allocation.

### Blinding

This study did not allow for the clinician or patients to be blinded. However, the researcher was blinded during both the measurement (data collection) and statistical analysis stages.

### Interventions

All patients were treated by a single researcher (Ş.A.) using a fixed, preadjusted edgewise appliance (0.018-in. Roth prescription, American Orthodontics, Mini Master, USA). Vertical slot brackets (AO Mini MS Max 020×020 V-slot, USA) were preferred for canine teeth. Archwire sequences were determined to be 0.014, 0.016, 0.016×0.016, 0.016×0.022, 0.017×0.025-in. nickel titanium (NiTi), respectively. Each archwire was used for 4 weeks, but only three patients required more than 4 weeks of a specific wire in the sequence for the relief of crowding. After 4 weeks of using 0.017×0.025 NiTi, 0.016×0.022 stainless steel (SS) archwire was installed, and the retraction phase was initiated. To minimize friction during retraction, a transition from 0.017×0.025-in. NiTi to 0.016×0.022-in. SS was undertaken [16, 17].

A 1.6 mm (diameter) × 8 mm (length) titanium miniscrew (DewiMed, Germany) was inserted intraradicularly between the upper second premolar and the upper first molar on each side for 3 months prior to the retraction phase. The miniscrews were ligated to the upper second premolar using 0.010-in. stainless steel wire for indirect anchorage. The first premolars were extracted by a single surgeon on the day the miniscrews were placed. The miniscrews were assessed for stability during monthly appointments. One patient was excluded from the study due to miniscrew failure.

**Fig. 1:**
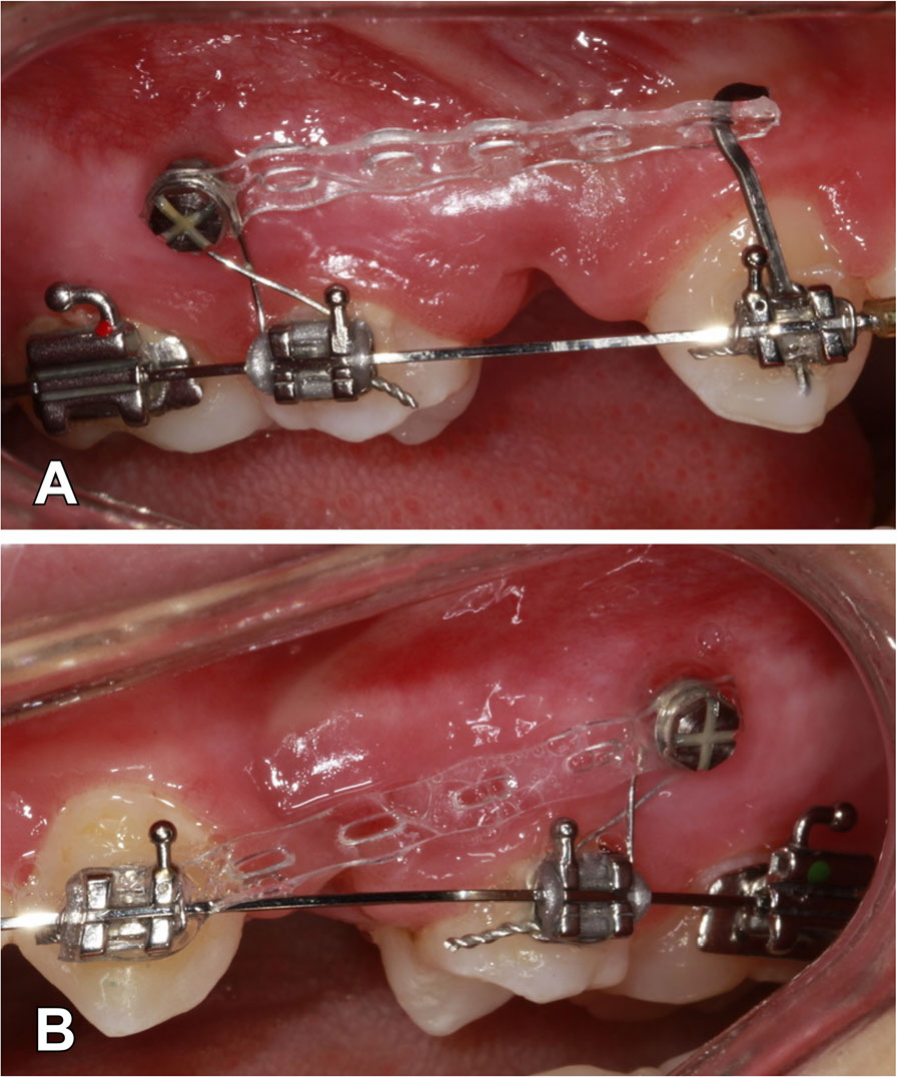
**A** Power arm sliding mechanic. **B** Direct sliding mechanic

The canine retractions were initiated 3 months after the teeth extraction. The power arm application was randomly selected either to the right or left side with a 1:1 allocation ratio using sealed, opaque envelopes. Each patient was asked to select previously shuffled envelopes. A power arm bent from 0.016×0.022-in. titanium molybdenum alloy (TMA) wire was placed in the vertical slot of the canine bracket on the randomly selected side. The length of this power arm was customized in accordance with the definition of the center of resistance in Nanda and Tosun’s book, which is nearly one-third of the root length [18]. Power arm length was determined using the ratio/proportion method on a panoramic radiograph. The elastomeric chain (Ortho Technology, USA) was applied from the power arm to the miniscrew on one side and from the canine bracket to the miniscrew on the other side (Fig. 1). The net force for each side was set at 150 g by using a tension gauge (Loyka Dial 0–500gf) [3, 15].

**Fig. 2:**
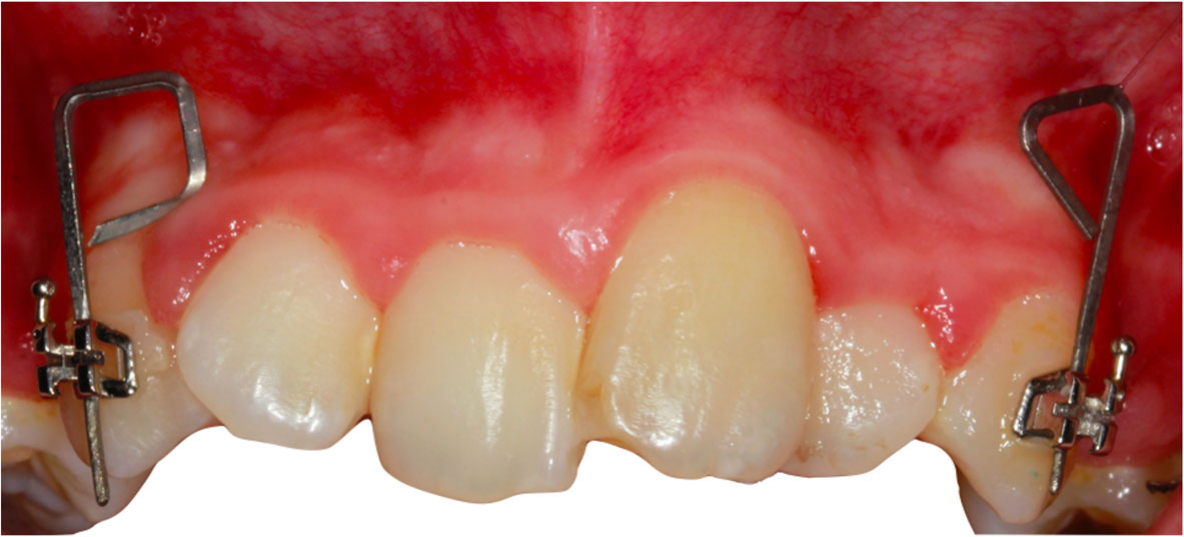
Metal jigs for angulation measurements

At the beginning of retraction (T0) and in the first month (T1), second month (T2), and third month (T3) of retraction, digital models were acquired with an intraoral TRIOS scanner (3Shape, Copenhagen, Denmark). Archwires were removed before the acquisition of the digital models, and the intraoral scanner was calibrated prior to each scan. To measure the angulation, custom metallic jigs (0.016×0.022-in. TMA wire) were inserted into the vertical slot of the canine bracket before the T0 and T3 scans (Fig. 2).

**Fig. 3:**
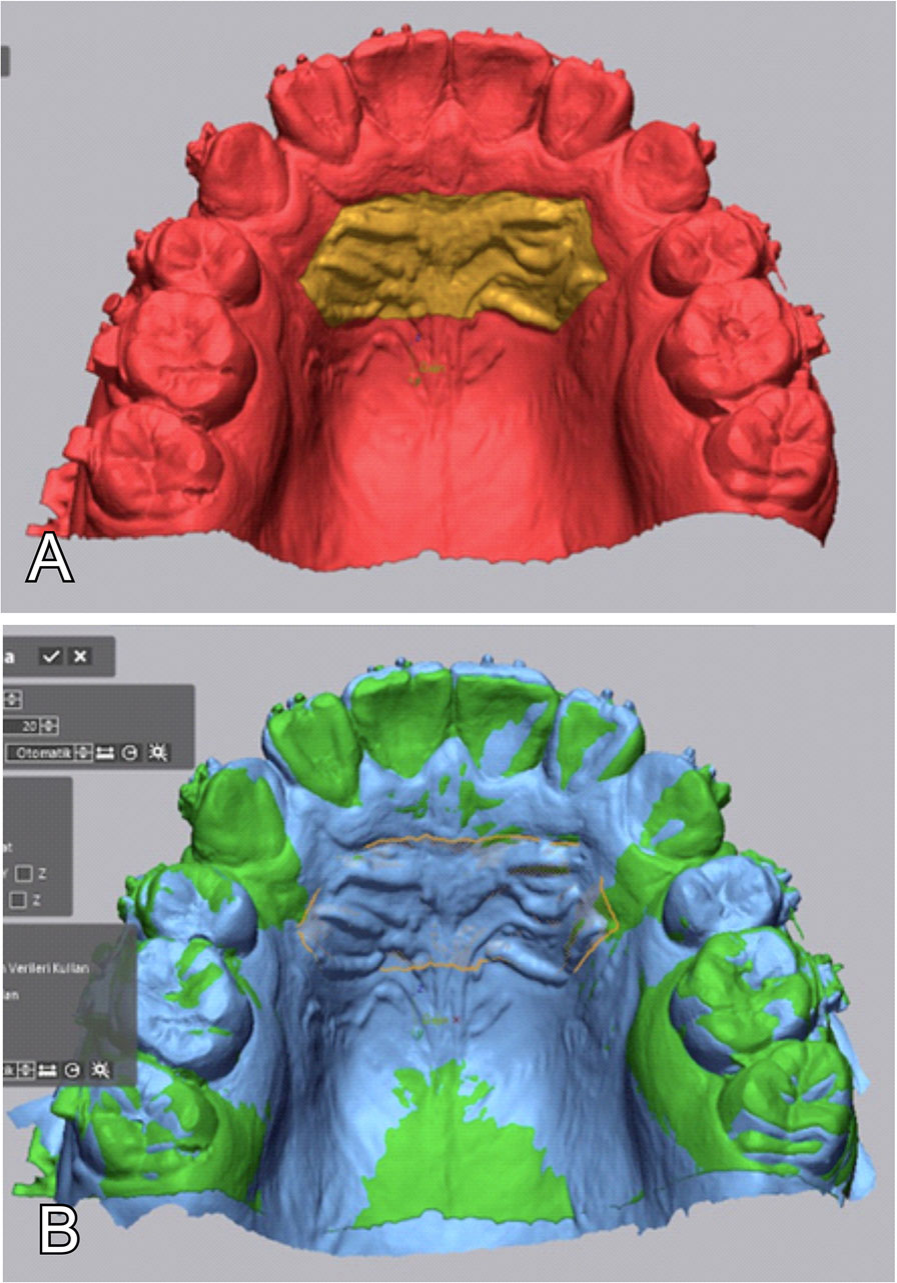
**A** Reference area selection. **B** Local best-fit superimposition

Using the Geomagic Control X (3D Systems, Rock Hill, SC) software, the four models obtained from each patient were separately superimposed on each other. The area between the lateral tips of the first and third rugae was chosen as the reference area for the superimpositions [15] (Fig. 3).

**Fig. 4:**
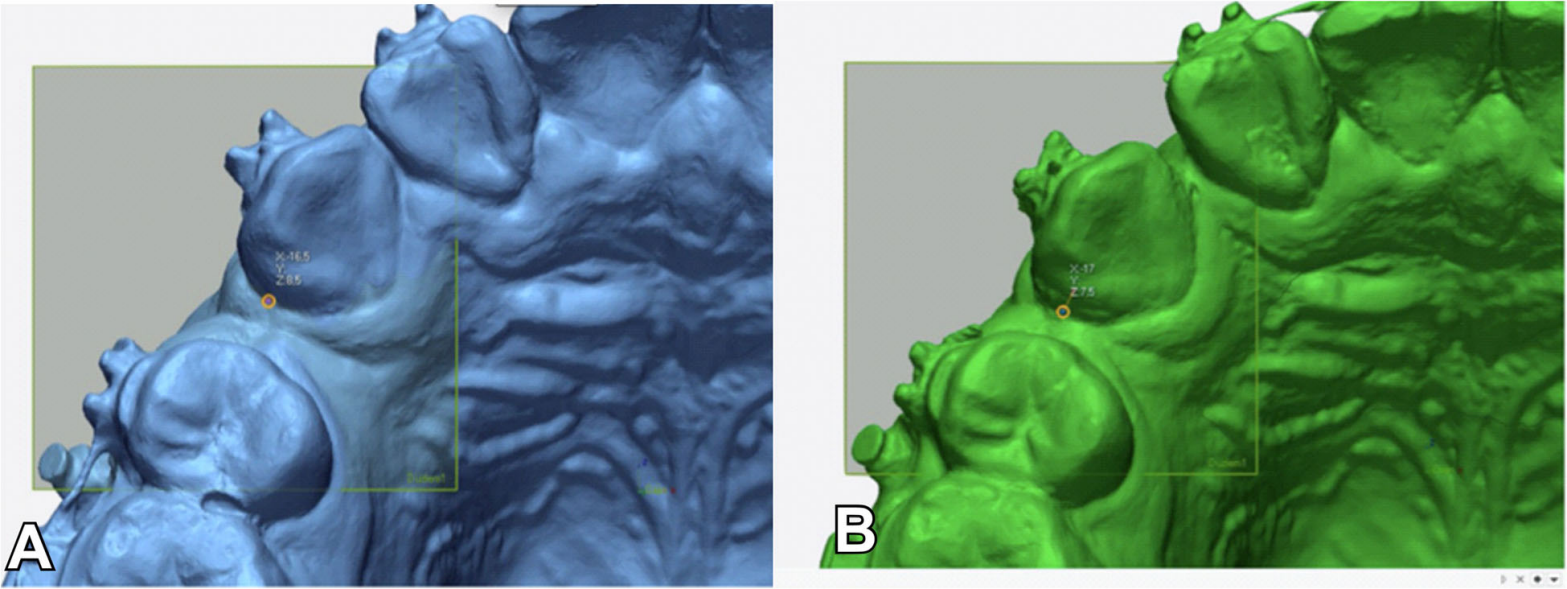
The calculation of the retraction rate using the horizontal plane. **A** Marking the most distal point of the canine in the T0 model. **B** Marking the most distal point of the canine in the T3 model

To calculate the retraction rate, the digital models were first superimposed, and a horizontal plane parallel to the occlusal plane was then created. For each model, the distal point of the canine teeth was marked on the plane, and the distance between the two points was calculated (Fig. 4). The retraction rate was analyzed monthly.

**Fig. 5:**
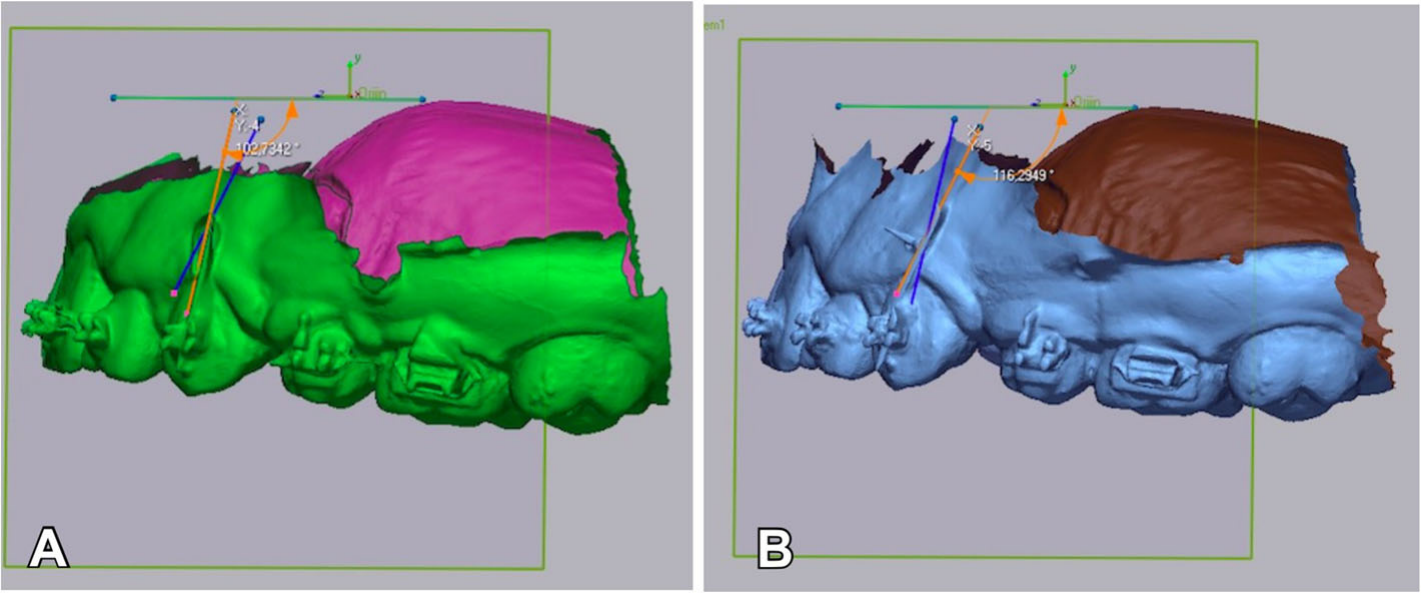
The calculation of angulation changes using metal jigs on the vertical plane. **A** Angulation measurement in the T0 model. **B** Angulation measurement in the T3 model

In the angulation measurements, a vertical plane was created that passed through the midpalatal suture while the models were in lateral view. A horizontal reference line was placed on the plane. The change in the angulation was determined by measuring the angle between the reference line and the line passing through the metal jigs (Fig. 5). Only total angulation change (T0–T3) was calculated at the end of the 3-month retraction.

**Fig. 6:**
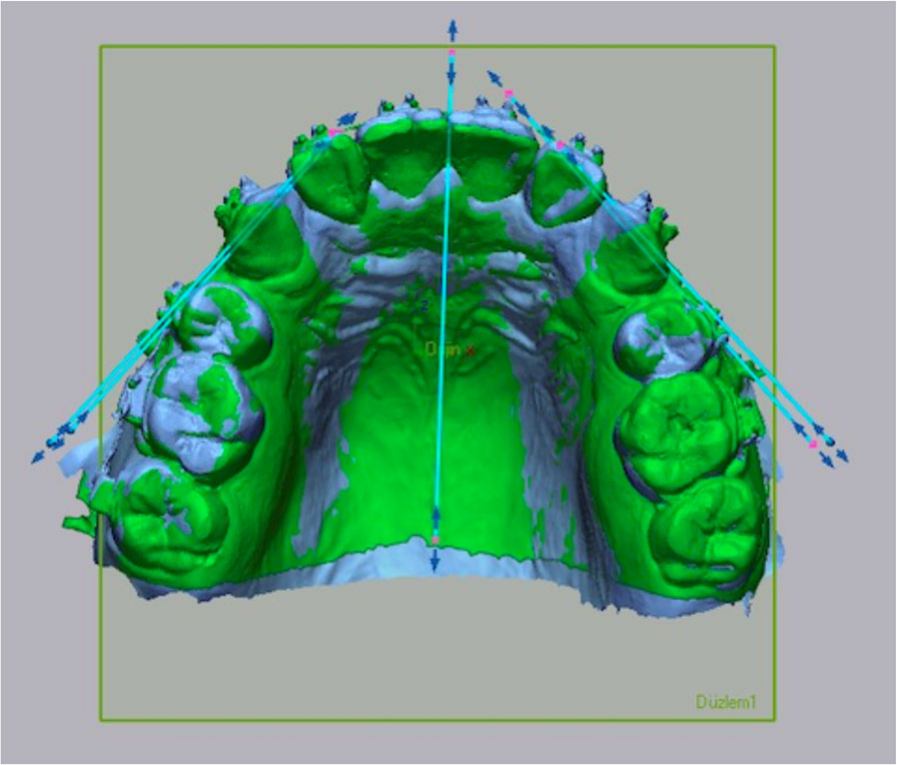
Method of rotation measurement

The Ziegler and Ingervall methods were adapted to digital model measurements to calculate rotation change [16, 19]. After the superimposition of the models, a horizontal plane parallel to the occlusal plane was created. A reference line was drawn on the surface of the plane along the midpalatal suture. The angle between the reference line and the line passing through the mesial and distal contacts of the canine was measured (Fig. 6). Only the total rotation change (T0–T3) was calculated.

The anchorage loss was measured by marking the most mesial point of the second premolar by following a similar procedure to that of the retraction rate measurements. Only the total anchorage loss (T0–T3) was calculated.

Specific angular and linear measurements were performed on the pre-treatment cephalometric radiographs using the AudaxCeph Version 5.X software (Ljubljana, Slovenia).

### Objectives (primary and secondary)

The primary objective of this study was to compare PAS and DS techniques in terms of retraction rate, anchorage loss, rotation, and angulation change. The secondary objective was to analyze the correlation between the retraction rate and gender or age for both retraction techniques.

### Interim analyses and stopping guidelines

Not applicable.

### Statistical analysis

Descriptive statistics, including mean values and standard deviations, were calculated. The paired-sample *t*-test and the Wilcoxon signed-rank test were used to compare changes in the T0–T1, T1–T2, and T2–T3 time intervals. The digital model measurements of eight patients were repeated 2 weeks later by the same researcher. An intraclass correlation test was used to analyze intra-examiner variability (Table 1). The values for female and male participants were compared with the Student’s *t*-test and the Mann–Whitney *U* test. Also, the correlation coefficients between age and other parameters were calculated. A *p* value <.05 was considered statistically significant.

**Table 1:** Intraclass correlation test results

Measurements	Retraction rate	Rotation	Angulation	Anchorage loss
Correlation coefficient	0.891	0.934	0.960	0.832

## Results

### Participant flow

**Fig. 7:**
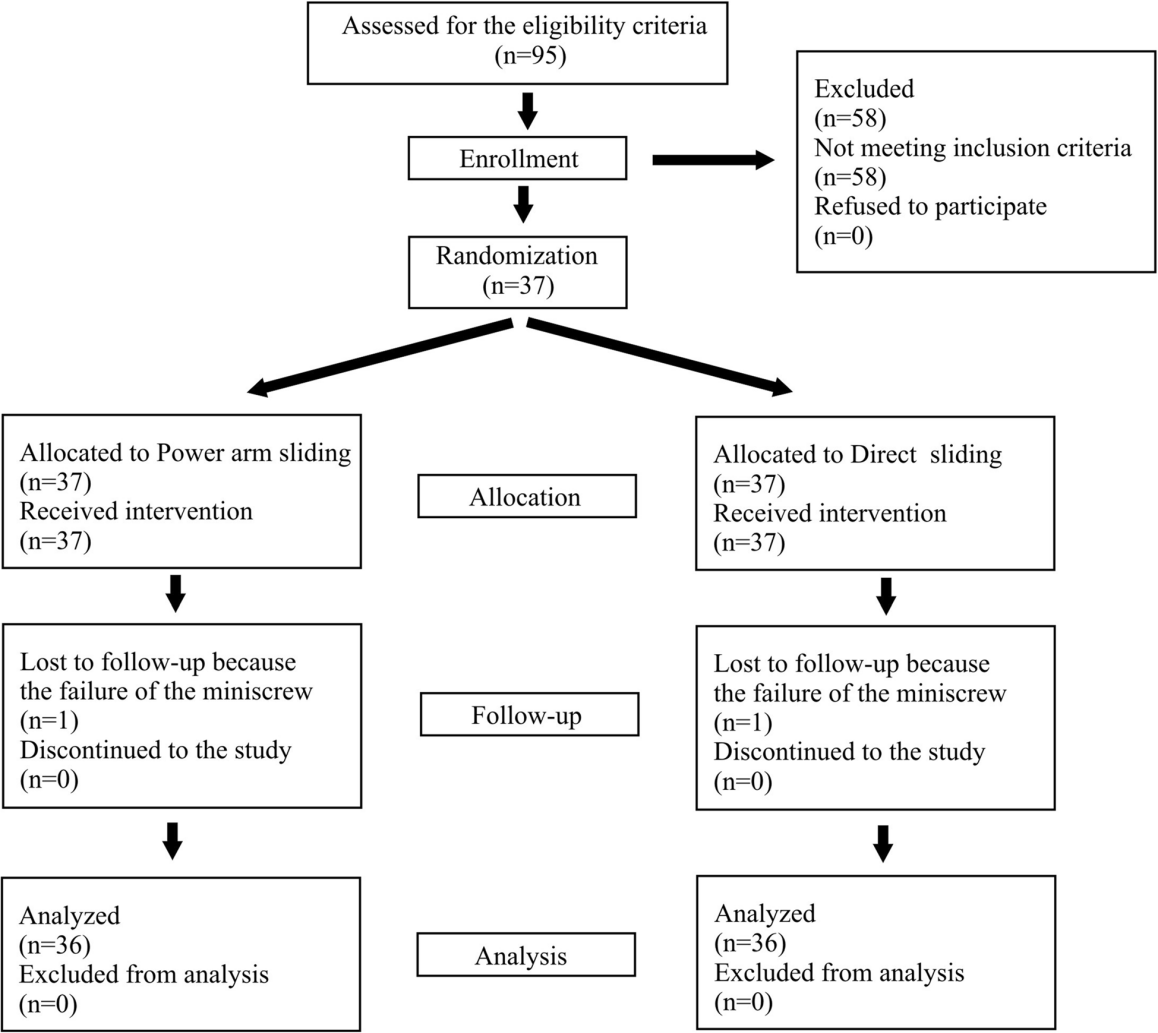
CONSORT flow chart

The study was conducted on 37 patients (20 females, 16 males; mean age, 16.94 ± 3.23 years). Only one patient was excluded due to a miniscrew failure. The flow chart of the study is shown in Fig. 7.

### Baseline data

The age range of the total sample was 13–29 years (mean 16.94 ± 3.23). The pre-treatment cephalometric values of the patients are shown in Table 2.

**Table 2:** Initial skeletal, dental, and demographic characteristics of the subjects

	***n*** (%)	Mean±SD
Gender		
Male	16 (45%)	
Female	20 (55%)	
Age (y)		16.94 ± 3.23
SNA (°)		81.2 ±4.2
SNB (°)		76.5 ±3.7
ANB (°)		4.5± 2.1
GoGn-SN (°)		32.6± 6.8
PP-MP (°)		25.2± 6.3
U1-SN (°)		104.9± 8.7
IMPA (°)		96.3 ±7.0
Overjet (mm)		5.3 ±2.5

### Numbers analyzed for each outcome

**Fig. 8:**
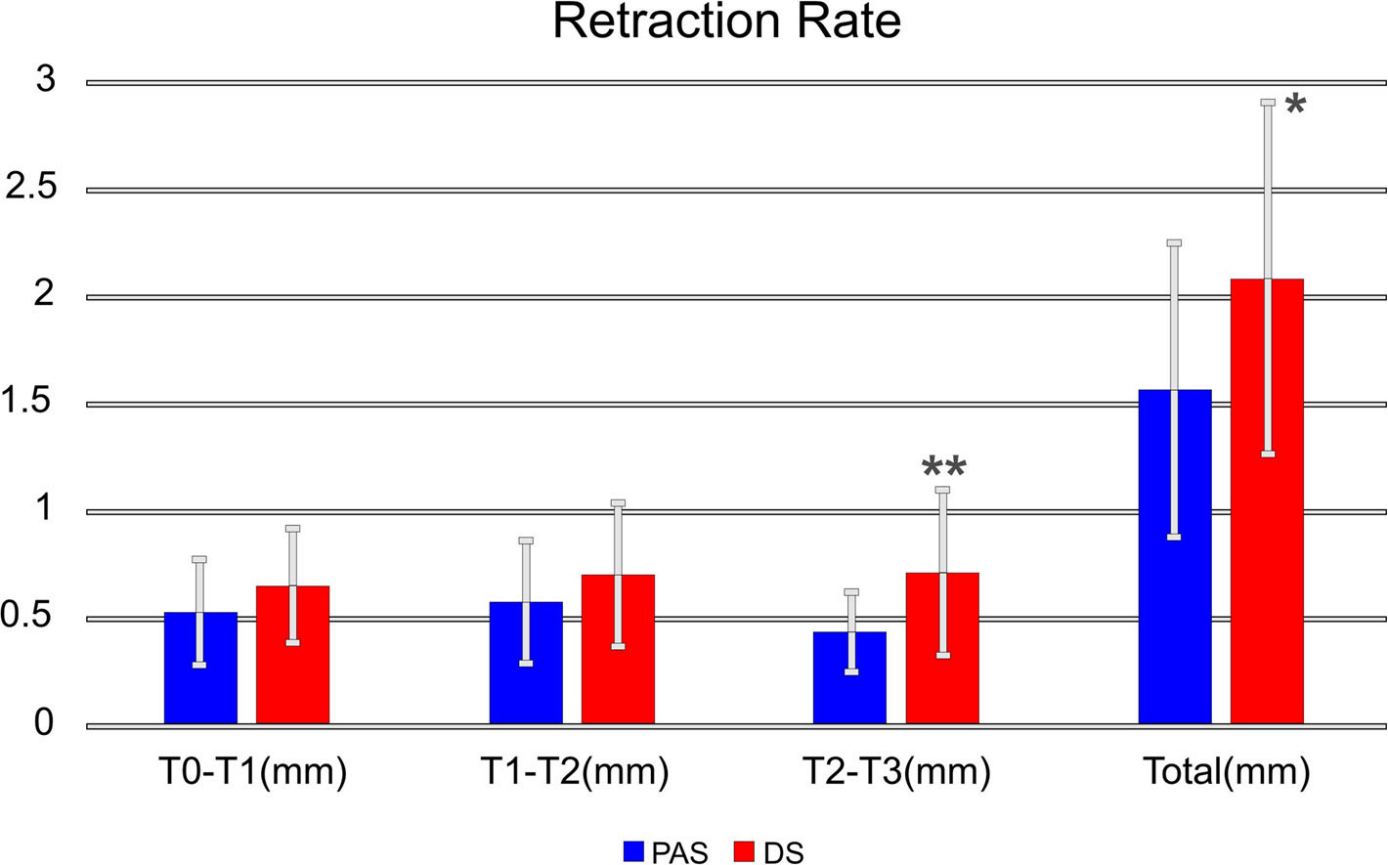
Changes in monthly retraction rate. PAS, power arm sliding, DS, direct sliding, * *p* < 0.05 ** *p* < 0.01

The retraction rates for the first 2 months (T0–T1 and T1–T2) showed no significant differences between groups (Fig. 8). The retraction rate for DS was significantly higher in the third month (T2–T3) compared to PAS (Table 3). The total retraction rate was also found to be higher in DS than in PAS.

**Table 3:** A comparison of monthly retraction rates of the two techniques

	PAS Mean (mm) ± SD	DS Mean (mm) ± SD	*p* value
T0–T1	0.53±0.26	0.66±0.28	0.050^a^
T1–T2	0.58±0.30	0.71±0.35	0.101^a^
T2–T3	0.44±0.20	0.72±0.39	0.000^b^**
Total	1.57±0.69	2.09±0.84	0.006^a^*

*PAS* power arm sliding, *DS* direct sliding

*p <0.05, **p <0.01

^a^Independent sample *t*-test

^b^Mann-Whitney *U* test

There were no statistically significant differences in angulation, rotation, and anchoring loss between the two techniques at the end of the 3-month retraction period (Table 4).

**Table 4:** A comparison of the 3-month angulation, rotation, and anchorage loss changes of the PAS and DS

	PAS Mean ±SD	DS Mean±SD	*p* value
Angulation (°)	3.62±2.91	4.82±3.08	0.094
Rotation (°)	7.57±4.70	8.49±5.25	0.443
Anchorage loss (mm)	0.35±0.32	0.33±0.26	0.786

*PAS* power arm sliding, *DS* direct sliding

*p*<0.05

The rate of retraction was independent of gender for both techniques (Table 5). A negative correlation between age and retraction rate was observed (the correlation coefficient for PAS −303 and for DS −169). However, this correlation was not statistically significant (*p* > 0.05).

**Table 5:** Comparison of the total amount of retraction by gender

	Female Mean ±SD	Male Mean±SD	*p* value
PAS (mm)	1.58±0.71	1.56±0.71	0.938
DS (mm)	2.13±0.71	2.05±1.01	0.798

*PAS* power arm sliding, *DS* direct sliding

*p*<0.05

### Harm

No harm was observed in any participant during the trial.

## Discussion

### Main findings in the context of the evidence and interpretation

Canine retraction is an important part of the total treatment time in cases treated with upper first premolar extraction [20]. Therefore, it is essential to select the most suitable retraction mechanism. Canine retraction techniques are classified as frictional and non-frictional [4, 21]. The objective of frictional sliding mechanics is to achieve bodily movement using rectangular slots of edgewise brackets [22]. However, auxiliary mechanics, such as the power arm, are sometimes used because the force applied directly from the bracket may be inadequate to allow for bodily movement. Bodily retraction can therefore be achieved as the force passes through the resistance center. The purpose of this study was to compare the effects (rate, rotation, angulation, and anchorage loss) of different force application methods (below or through the center of resistance) in canine retraction by using three-dimensional digital model measurements.

Frictional retraction mechanics have disadvantages, such as the tipping of the canine, the restriction of movement because of a binding effect, the loss of anchorage, and the extrusion of incisors [23]. However, orthodontists often use these mechanics because of their easy application, the control of the entire dental arch with a single archwire, and thus less chair time [24]. In this study, instead of using a full-sized archwire during retraction, 0.016×0.022-in. steel was used to reduce friction [25, 26]. The elastomeric chain was preferred to the nickel-titanium coil spring, which produces a continuous retraction force [3, 27]. The reason for this preference was that the force degradation of the elastomeric chains allow for the uprighting of the canine during retraction. When using a closed spring coil, severe tipping of the canine would be observed, and the results would be adversely affected.

Buchmann et al. reported that a significant force degradation of the elastomer chain occurred in the first 24 h [28]. Hasler et al. suggested that the canine retraction rate on the healing side was lower than on the recent extraction site [29]. The monthly retraction rate for this study was relatively low compared to previous studies, possibly because of its using elastomeric chains and starting canine retraction 3 months after premolar extraction. However, the amount of retraction was within the range of monthly anterior–posterior movements (0.35–2.04 mm) reported by Norman et al. [30]. The total retraction rate of DS was higher than that of PAS. According to the authors, this was due to bodily movement being clinically difficult. Shpack et al. reported similar findings in their study [16].

In similar studies analyzing canine retraction mechanics, a wide range of ages has been preferred in sample selection [21, 31, 32]. In Dinçer and İşcan’s study, the average age of the participants was 13.7 [31]. Alkebsi et al. included only patients older than 16 years of age in their study [15]. Age could affect the retraction rate by different levels of bone maturation [33]. However, in this split-mouth design study, age did not negatively affect the findings. The results revealed that the negative correlation between age and retraction rate was not statistically significant.

Chisari et al. suggested that retraction rates were different for male or female patients [34]. However, Dudic et al. reported that orthodontic tooth movements were independent of gender factors [35]. Similarly, the findings of the present study showed that gender was not a factor that affects the retraction rate.

Previous studies have investigated applications that increase the inflammatory response, such as micro-osteoperforation or corticotomy, to accelerate tooth movement [25, 36]. To minimize the inflammatory response that occurs immediately after tooth extraction (the regional acceleration phenomenon [37]), retraction began 3 months after tooth extraction. Leethanakul et al. reported that 3 months was sufficient for bone maturation in the extraction socket [3]. The 3-month waiting period may have reduced the monthly retraction rate, though.

### Limitations

The comparison of only the short-term (a 3-month follow-up period) effects of PAS and DS methods was a limitation of this study. Another limitation was that there was no questionnaire to assess pain levels and patient satisfaction. Further studies are necessary to examine the long-term effects of these two mechanics.

### Generalizability

The study’s findings revealed that the DS method provides faster retraction. In terms of unwanted tooth movements (tipping, rotation, and anchorage loss), there was no significant difference between the two methods. However, because the retraction lasted only 3 months and was conducted by a single clinician on a limited group of patients, the results could not be generalized.

## Conclusions

- The direct sliding retraction rate was higher than the power arm sliding.
- There was no significant difference between the two methods in terms of anchor loss, rotation, and angulation change.
- The retraction rate was independent of gender and age.
- Miniscrews were successful in preventing anchorage loss.

## Data Availability

Data and materials are available at the Orthodontic Department in the Faculty of Dentistry, Afyonkarahisar Health Science University.
